# Scientific Items

**Published:** 1887-05

**Authors:** 


### SCIENTIFIC ITEMS.

**Rroving the Soundness of an Eye.**—In a large factory in which were employed several hundred persons, one of the workmen, in wielding his hammer, carelessly allowed it to slip from his hand. It flew half way across the room, and struck a fellow workman in the left eye. The man averred that his eye was blinded by the blow, although a careful examination failed to reveal an injury, there being not a scratch visible. He brought a suit in the courts for compensation for the loss of half of his eyesight, and refused all offers of compromise. Under the law, the owner of the factory was responsible for an injary resulting from an accident of this kind; and although he believed the man was shamming, and that the whole case was an attempt at swindling, he had about made up his mind that he would be compelled to pay the claim. The day of the trial arrived, and in open court an eminent oculist retained by the defense examined the alleged injured member, and gave his opinion that it was as good as the right eye. Upon the plaintiff's loud protest of his inability to see with his left eye, the oculist proved him a perjurer, and satisfied the court and jury of the falsity of his claim. And how do you suppose he did it? Why, simply by knowing that the colors green and red combined make black. He prepared a black card on which a few words were written with green ink. Then the plaintiff was ordered to put on a pair of spectacles with two different glasses, the one for the right eye being red and the one for the left consisting of ordinary glass. Then the card was handed him, and he was ordered to read the writing on it. This he did without hesitation, and the cheat was at once exposed. The sound right eye, fitted with the red glass, was unable to distinguish the green writting on the black surface of the card, while the left eye, which he pretended was sightless, was the one with which the reading had to be done.—*Pottery Gazette.*

**Her Schiller**, a well known German architect, reports some facts which are of interest, as indicating the radius of the circle of protection of good lightning rods. On June 17 last, at the village of Mottingen, lightning struck a pear tree 33 feet high. On one side, 115 feet away, was a schoolhouse, with a rod 6 feet high. On the other side was a church, 328 feet away, and having a lightning rod reaching up 154 feet. Both rods are well placed, and had worked well when tested, and the level of the foot of the tree is about the same as that of the two buildings. It is evident, then, if the facts have been accurately reported, that the radius of the circle of protection is not more than twice the height of the rod.—*Scientific American.*

**Professional Fatigue.**—Medical and other professional men often break down from their inability to keep a regular time for meals. An eminent doctor says:

"Being often out for many hours, and becoming too exhausted to digest a full meal when at length able to get it, I conceived a plan which answered admirably well, and which other doctors have gladly adopted. I provided myself with a small bottle of lime water, which I add to a glass of milk when passing a dairy shop; or I put a small flask of the mixture in my pocket. A water biscuit with this will keep a man harmless on a long fast, and enable him to digest a meal when he can obtain one."—*Scientific American.*

**Vegetable Meat-Eaters.**—One of the best known of these insect-eating plants found here, as well as in Lapland and Scandinavia, is the Sun-dew (*Drosera*), discovered about a century ago. Another plant, the so-called Flytrap of Venus (*Dionæa*) of America, which was brought to England 120 years ago, has received the name of Venus, for the reason that, like the goddess of beauty it attracts and captivates everything that heedlessly approaches it. At the bottom of the plant the leaves cluster like a rosette; from the center of this arises the flower stalk. The edge of the leaf, which is nearly circular, is overgrown with strong bristles, while its surface is covered with small glands, at either side of which are three long hairs. A fly approaches; carelessly it settles on the leaf, and perchance touches one of the six long hairs. Suddenly the leaf folds, the bristles interlace, and the insect is caught. Oftentimes the whole tragedy takes but ten seconds. The sensitive hairs have performed their duty; now begins the work of the glands. These discharge a large quantity of colorless acid slime—the digestive fluid, pepsin—and the closed leaf changes at once into a stomachic organ. After a lapse of eight or nine days the leaf reopens—the insect has disappeared—the prey has been consumed. The above-mentioned facts constitute the main features of the process of digestion, but in connection with it many questions arise. What happens, for instance, if a nonedible object irritate the hairs, perhaps a stone or a piece of wood? The leaf closes with the greatest possible swiftness, but soon discovers its mistake, and does not discharge the digestive juice; after a lapse of twenty-four hours it again unfolds, ready for anothe capture. This does away with the mark of distinction thus far generally accepted, namely, that "plants live, animals live and feel" (*plantæ vivunt, animalia vivunt et sentiunt*), for the *Dionæa* distinguishes quite readily, by taste and feeling, that which is digestible from that which is not. By experiment, it has been ascertained that nitrogenous nourishment is preferred by the *Dionæa;* hence every kind of meat (beef, pork, and veal, either raw, fried, or stewed) is digested by the plant, also albumen and cheese; the latter, however, causes disturbances during digestion, and the leaf easily ails.—*Popular Science Monthly.*

**A Pocket Corkscrew** has been patented by Mr. Leroy B. Haft, of Englewood, N. J. This invention relates to a corkscrew wherein the screw is pivoted to a carrier sliding within the handle. A good thing for a doctor.

